# Körperliche und psychische Folgeerkrankungen bei Diabetes mellitus

**DOI:** 10.1007/s00103-022-03517-y

**Published:** 2022-03-16

**Authors:** Bernhard Kulzer

**Affiliations:** 1 Forschungsinstitut der Diabetes-Akademie Bad Mergentheim (FIDAM), Theodor-Klotzbücher-Str. 12, 97980 Bad Mergentheim, Deutschland; 2 Diabetes-Klinik Bad Mergentheim, Bad Mergentheim, Deutschland; 3 Lehrstuhl für klinische Psychologie, Universität Bamberg, Bamberg, Deutschland

**Keywords:** Mikroangiopathie, Makrangiopathie, Depressionen, Lebensqualität, Mortalität, Microangiopathy, Macroangiopathy, Depression, Quality of life, Mortality

## Abstract

Trotz Verbesserungen in der Therapie des Diabetes und besseren Versorgungbedingungen weisen die Betroffenen aktuell im Vergleich zur Allgemeinbevölkerung noch immer ein deutlich erhöhtes Risiko für physische wie psychische Folgeerkrankungen sowie eine reduzierte Lebensqualität auf. Etwa 21 % aller Todesfälle sind in Deutschland auf Diabetes und seine Folgeerkrankungen zurückzuführen, das Mortalitätsrisiko ist für Menschen mit Diabetes um mehr als das 1,5-Fache gegenüber Menschen ohne Diabetes erhöht. In dieser Übersicht werden die Verbreitung und die Risikofaktoren für die häufigsten körperlichen und psychischen Folgen des Diabetes beschrieben sowie deren Einflüsse auf die Lebensqualität der Patienten. Zusammenhänge zwischen den Folgeerkrankungen und einer erhöhten Mortalität werden aufgezeigt.

In großen Interventionsstudien konnte die Bedeutung einer guten Glukoseeinstellung – vor allem zu Beginn der Erkrankung – in Hinblick auf eine Senkung der Mortalitätsrate gezeigt werden, weitere wichtige Einflussfaktoren sind z. B. Blutdruck, Blutfette und Rauchen. Weltweite Studienergebnisse deuten auf einen stabilen Trend hinsichtlich einer verbesserten Lebenserwartung von Menschen mit Diabetes in den letzten Jahren hin. Zukünftig könnte der positive Trend durch bessere Versorgungsstrukturen und neue Technologien sowie digitale Anwendungen in der Forschung und Therapie fortgesetzt werden. Mithilfe der Präzisionsmedizin könnten individuelle Risikofaktoren und protektive Faktoren erkannt werden, um der Entstehung von Folgekomplikationen noch besser vorzubeugen.

## Abstract (en)

Despite improvements in the treatment of diabetes and better care conditions, those affected still have a significantly increased risk of physical and psychological complications and a reduced quality of life compared to the general population. About 21% of all deaths in Germany are due to diabetes and its complications, and the mortality risk for people with diabetes is more than 1.5 times higher than for people without diabetes. This review describes the prevalence and risk factors for the most common physical and psychological complications of diabetes as well as their influences on patients’ quality of life. Associations between the complications and increased mortality are shown.

In large intervention studies, the importance of good glucose control – especially at the beginning of the disease – has been shown regarding a reduction in the mortality rate. Other important influencing factors include blood pressure, blood lipids, and smoking. Worldwide study results indicate a stable trend about improved life expectancy of people with diabetes in recent years. In the future, the positive trend could be continued through better care structures and new technologies as well as digital devices in research and therapy. With the help of precision medicine, individual risk factors and protective factors could be identified to even better prevent the development of complications.

## Einleitung

Diabetes mellitus ist eine häufig vorkommende Stoffwechselerkrankung. In Deutschland leben 8,5 Mio. Menschen mit Typ-2-Diabetes, wobei dieser bei schätzungsweise weiteren 2 Mio. Betroffenen bisher nicht diagnostiziert wurde. 32.000 Kinder und Jugendliche sowie 341.000 Erwachsene haben einen Typ-1-Diabetes und über 50.000 Frauen einen Schwangerschaftsdiabetes (Gestationsdiabetes; [1]). Bei Typ-2-Diabetes und Gestationsdiabetes sind die wichtigsten Therapiemaßnahmen eine Veränderung des Lebensstils, die Einnahme oraler Antidiabetika oder eine Inkretin- bzw. Insulininjektionstherapie, Menschen mit Typ-1-Diabetes substituieren das fehlende Hormon Insulin durch eine Form der Insulintherapie.

Für Menschen mit Diabetes stellt die Erhaltung der Lebensqualität das wichtigste Ziel der Diabetestherapie dar [2]. Dies bedeutet, trotz und mit Diabetes physisch und psychisch weitgehend gesund zu bleiben, die eigenen Lebensziele verwirklichen zu können und sozial integriert zu sein – ohne wichtige Einbußen in den für Menschen wichtigen Lebensbereichen, wie beispielsweise Familie/Beziehung, Beruf, Interessen oder Freizeit, zu erleiden. Um dieses Ziel zu erreichen, ist es vor allem wichtig, negative physische, psychische und soziale Folgen des Diabetes und eine diabetesassoziierte erhöhte Mortalität zu vermeiden. Dies gelingt aktuell leider nicht vollständig. Zudem ist die Lebenserwartung im Vergleich zur Allgemeinbevölkerung noch immer reduziert [3–9]. Da die körperlichen und psychischen Folgen des Diabetes schon sehr frühzeitig – manchmal sogar schon bei der Diagnose des Diabetes (z. B. Neuropathie, Retinopathie) – auftreten, wird nicht der Begriff „Langzeitfolgen“, sondern stattdessen „Folgeerkrankungen“ oder „‑komplikationen“ verwendet. Ziel dieses Artikels ist es, einen Überblick über die physischen wie psychischen Folgeerkrankungen sowie die Langzeitfolgen des Diabetes und die Lebensqualität von Menschen mit Diabetes zu geben.

## Folgekomplikationen: Bedeutung von psychologischen, verhaltensbezogenen und sozialen Faktoren

Für den Verlauf des Diabetes und das Risiko subsequenter Folgeerkrankungen spielen neben somatischen Faktoren auch psychologische, verhaltensbezogene und soziale Faktoren eine wesentliche Rolle [7, 8]. Dies ist vor allem der Tatsache geschuldet, dass Menschen mit Diabetes die wesentlichen Therapiemaßnahmen in ihrem persönlichen Alltag dauerhaft und selbstverantwortlich umsetzen müssen. Das Auftreten von Folgekomplikationen hängt zu einem hohen Ausmaß davon ab, wie gut ihnen dies langfristig gelingt.

**Figure 1:**
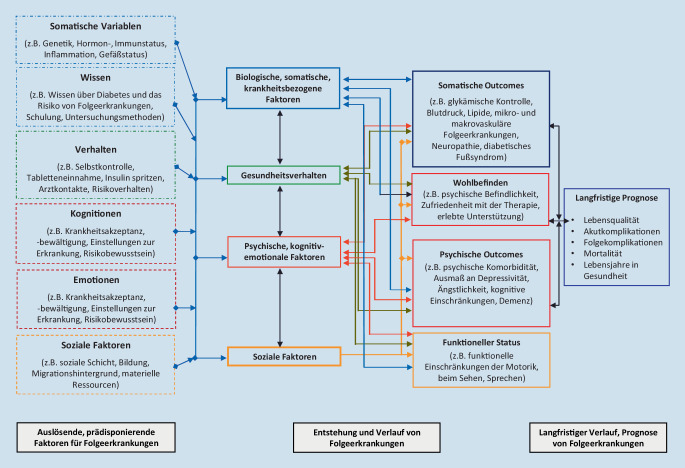

Heute können unterschiedliche somatische, kognitive, emotionale, verhaltensbezogene und soziale Variablen unterschieden werden, die entweder prädisponierende Faktoren für Folgeerkrankungen des Diabetes sind oder die Entstehung von Folgekomplikationen beeinflussen bzw. für den Verlauf und die langfristige Prognose eine bedeutsame Rolle spielen (Abb. 1). Interventionen, die auf die primäre, sekundäre und tertiäre Prävention von Folgeerkrankungen abzielen, müssen daher diese verschiedenen Einflussfaktoren mitberücksichtigen. Sie sollten nach Möglichkeit multidimensional und -disziplinär erfolgen. Dieses Modell gilt mit unterschiedlicher Gewichtung für alle Folgeerkrankungen des Diabetes und wurde z. B. für den diabetischen Fuß detaillierter beschrieben [10].

## Körperliche Folgen des Diabetes

Ein wesentliches Ziel der Diabetestherapie besteht in der Vermeidung von mikro- und makrovaskulären Folgeerkrankungen, Neuropathien und des diabetischen Fußsyndroms (DFS; [11]). Trotz erheblicher Erkenntnislücken bezüglich der Ätiologie, Therapie und Prognose der verschiedenen Komplikationen haben sich in den letzten Jahren deutliche Fortschritte in Hinblick auf komplexere pathophysiologische und therapeutische Ansätze ergeben. Im Sinne einer Präzisionsmedizin gelingt es – nicht zuletzt auch aufgrund des Einsatzes von Methoden der künstlichen Intelligenz – immer besser, Subtypen des Diabetes mit unterschiedlichen Risiken und Verläufen bezüglich Folgekomplikationen des Diabetes zu identifizieren [12]. In der Deutschen Diabetesstudie wurden beispielsweise mittels Clusteranalysen und künstlicher Intelligenz anhand der Variablen Alter, Body-Mass-Index, Glykämie, Homöostasemodellschätzungen und Inselautoantikörpern verschiedene Diabetesphänotypen vorgeschlagen, die eine unterschiedliche Prävalenz von Diabeteskomplikationen in frühen Stadien der nichtalkoholischen Fettlebererkrankung und der diabetischen Neuropathie prognostizieren [13].

### Retinopathie

Die diabetische Retinopathie ist nach wie vor eine der häufigsten Komplikationen des Diabetes und kann die gesamte Retina als auch die Makula betreffen. Weltweit wird die Prävalenz der diabetischen Retinopathie auf ca. 35 % geschätzt: Jeweils ca. 7,5 % entfallen auf die proliferative Retinopathie bzw. das diabetische Makulaödem und ca. 10 % auf eine visusbedrohende Retinopathie [14]. In Deutschland beträgt die Prävalenz der Retinopathie bei Typ-1-Diabetes ca. 24–27 %, bei Typ-2-Diabetes 9–16 %. Die Prävalenz der Makulopathie liegt bei Typ-1-Diabetes bei ca. 10 %, bei Typ-2-Diabetes bei ca. 6 % [15]. Die Erblindungsrate hat sich in den letzten 30 Jahren um ca. 75–80 % reduziert und liegt aktuell bei ca. 0,2–0,6 % [15]. Prognostisch sind neben einer längeren Diabetesdauer weitere Risikofaktoren bedeutsam: Hyperglykämie, Hypertonie, hormonelle Umstellung (Schwangerschaft, Pubertät), Nephropathie und bei Typ-1-Diabetes Rauchen und männliches Geschlecht [3]. Aufgrund der Symptomlosigkeit der Erkrankung im frühen Stadium sind Screeninguntersuchungen wesentlich, da die Prognose auch entscheidend von der Diagnosestellung in einem frühen Stadium und von einer ggf. notwendigen panretinalen Laserkoagulation abhängt.

### Nephropathie

Bei der diabetischen Nephropathie kommt es zu einer Veränderung der Eiweißausscheidung im Urin (progressive Albuminurie), zu einer Abnahme der glomerulären Filtrationsrate, einem erhöhten arteriellen Blutdruck bis hin zu einer terminalen Niereninsuffizienz. Weltweit ist Diabetes die häufigste Ursache für eine chronische Nierenerkrankung und Nierenversagen. Die Prävalenz der Nephropathie liegt in Deutschland bei Typ-1-Diabetes bei ca. 30 %, bei Typ-2-Diabetes bei ca. 40 % [4]. Mittlerweile mehren sich Befunde, dass der peripheren Insulinresistenz und den damit assoziierten inflammatorischen Prozessen eine bedeutsame pathophysiologische Rolle bei der Entstehung und Progression der Nephropathie zukommt. In 3 unabhängigen Studien konnte gezeigt werden, dass die Gruppe von Menschen mit Typ-2-Diabetes, die eine ausgeprägte periphere Insulinresistenz aufweist, ein besonders hohes Risiko für die Entwicklung und Progression einer Nephropathie hat [16]. Eine Studie im Zeitraum 2010 bis 2016, in der Daten von rund 25 Mio. Versicherten der Allgemeinen Ortskrankenkassen/Betriebskrankenkassen ausgewertet wurden, hat gezeigt, dass Menschen mit Diabetes 6‑fach häufiger eine Nierenersatztherapie bekommen als Menschen ohne Diabetes, wobei jedoch pro Jahr ein Rückgang der Nierenersatztherapie um 3 % zu verzeichnen war [17]. Die Progression der Niereninsuffizienz wird maßgeblich durch das Ausmaß der Albuminurie und die Einschränkung der Nierenfunktion bestimmt. In einem frühen Stadium sind die Veränderungen an den Glomeruli zumindest teilweise reversibel, wenn der Blutdruck, die Glukose und die Lipide optimal eingestellt werden und eine angemessene Therapie erfolgt [4].

### Neuropathie

Die diabetische Neuropathie ist eine Erkrankung der peripheren Nerven, welche als distal-symmetrische diabetische sensomotorische Polyneuropathie oder autonome Neuropathie auftreten kann. Klassifiziert wird sie entsprechend dem Befallsmuster, der Schmerzcharakteristika, der Organmanifestationen und der Beteiligung des autonomen Nervensystems [18]. Die Polyneuropathie tritt oft beidseitig an Füßen oder Fingern auf und kann brennende, einschießende oder stechende Schmerzen, Missempfindungen, Kribbeln oder Taubheitsgefühl umfassen. Die Prävalenz liegt bei Personen mit Typ-1- und Typ-2-Diabetes bei ca. 30 %, ca. 13–26 % weisen eine schmerzhafte Neuropathie auf. Personen mit einer sensomotorischen Polyneuropathie, die mit eingeschränktem Schmerzempfinden einhergeht, sind eine spezielle Risikogruppe hinsichtlich Fußläsionen und der Entwicklung eines diabetischen Fußsyndroms. Mehr als 90 % der Betroffenen mit einem diabetischen Fußsyndrom weisen eine sensible Polyneuropathie auf [19]. Die Therapie der diabetischen Neuropathie besteht aus einem multimodalen, risikoadaptierten Behandlungsansatz mit nichtmedikamentösen und medikamentösen Ansätzen, wobei die Güte der Glukoseeinstellung bei der Therapie oft nur eine untergeordnete Rolle spielt [19].

### Periphere arterielle Verschlusskrankheit (pAVK)

Etwa 40 % aller Patienten in Deutschland mit einer pAVK – einer peripheren Durchblutungsstörung, welche Stenosen, Verschlüsse und aneurysmatische Gefäßveränderungen der Becken-Bein-Arterien umfasst – sind an Diabetes erkrankt. Bei Personen mit Diabetes über 70 Jahren beträgt die Prävalenz ca. 20 %. Zudem entwickelt sich bei Menschen mit Diabetes die pAVK früher, weist eine höhere Progressionsrate auf und geht häufiger in die kritische Extremitätenischämie über [20]. Das relative Risiko für das Auftreten einer pAVK in Verbindung mit Diabetes im Vergleich zu Menschen ohne Diabetes liegt bei Frauen bei 1,96 (95 % KI 1,29–2,63) und bei Männern bei 1,84 (95 % KI 1,29–2,86), sodass Diabetes neben Rauchen und hohem Alter der bedeutsamste Risikofaktor der pAVK ist [21]. Die wichtigsten Folgen der pAVK sind Fußläsionen (Ulzerationen, Gangrän) und aufgrund des ischämischen oder neuroischämischen diabetischen Fußsyndroms (DFS) Minor- und Majoramputationen. Die Therapie der pAVK zielt darauf ab, den peripheren Blutfluss bei symptomatischen Patienten zu verbessern und vaskuläre Risikofaktoren und Begleiterkrankungen unter besonderer Berücksichtigung koronarer und zerebrovaskulärer Gefäßerkrankungen zu behandeln [22]. Die Therapieergebnisse nach Revaskularisierungsverfahren wie der perkutanen transluminalen Angioplastie und Bypässen sind bei Menschen mit Diabetes schlechter, ebenfalls die perioperative Morbidität und Mortalität [21].

### Diabetisches Fußsyndrom (DFS)

Die Neuropathie und die pAVK zählen zu den wichtigsten prädisponierenden Faktoren des DFS, Traumata und Infektionen zu den Hauptauslösern. Das Nichtheilen eines Ulkus oder eine Amputation hängt von einer Vielzahl unterschiedlicher somatischer Faktoren ab, deren Wechselwirkung noch nicht gänzlich verstanden ist [23]. Die Wahrscheinlichkeit eines DFS beträgt für die gesamte Lebensdauer eines Menschen mit Diabetes 19–34 %, die jährliche Neuerkrankungsrate liegt bei ca. 2 %. 65–70 % aller Fußamputationen werden bei Menschen mit Diabetes mellitus durchgeführt [24]. Für den Zeitraum von 2008–2012 (Durchschnittsalter 72,6 Jahre) konnte in Deutschland ein signifikanter Rückgang von Majoramputationen pro 100.000 Personenjahren von 81,2 auf 58,4 sowie leichter Amputationen von 206,1 auf 177,0 beobachtet werden [25]. Die wichtigsten Risikofaktoren für Fußulzerationen bei Menschen mit Diabetes sind ein fehlendes Druckempfinden (Test mit 10-g-Monofilament), mindestens ein fehlender Fußpuls, eine längere Diabetesdauer sowie eine Vorgeschichte von Ulzerationen [6]. Weibliches Geschlecht ist hingegen ein protektiver Faktor. Insgesamt ist die Prognose bei Vorliegen des DFS eher schlecht, da die kumulative Rezidivrate bei ca. 70 % liegt, die 5‑Jahres-Überlebensrate nach Auftreten eines neuen Ulkus nur bei ca. 50–60 % [26].

### Herzerkrankungen

Makroangiopathische Veränderungen sind vor allem bei Menschen mit Typ-2-Diabetes sehr ausgeprägt, sodass diese ein deutlich erhöhtes Risiko für die Entwicklung kardiovaskulärer Erkrankungen wie akuter Myokardinfarkt, Schlaganfall und kardiovaskulärer Tod aufweisen. Ein 60-jähriger Mann mit Diabetes und einem Herzinfarkt verliert im Vergleich zur Allgemeinbevölkerung ca. 12 Lebensjahre [27]. Das kardiovaskuläre Risiko erhöht sich bereits im Stadium des Prädiabetes, da sich häufig bereits in jungen Jahren eine endotheliale Dysfunktion nachweisen lässt, die eng mit einer Insulinresistenz assoziiert ist. Personen mit Diabetes weisen häufiger als Menschen ohne Diabetes Vorhofflimmern auf und sind besonders durch thrombembolische Komplikationen gefährdet. Auch das Risiko einer Herzinsuffizienz ist für Menschen mit Diabetes im Vergleich zu Menschen ohne Diabetes verdoppelt [28]. Daten der PARADIGM-HF-Studie zeigen über 27 Monate eine um 17 % erhöhte kardiovaskuläre Mortalität bei Patienten mit Herzinsuffizienz und Diabetes im Vergleich zu Menschen ohne Diabetes. Innerhalb eines Jahres liegt für Personen mit Diabetes das Risiko für eine Hospitalisierung wegen Herzinsuffizienz oder für den kardiovaskulären Tod zwischen 12 % und 15 % [29]. Menschen mit Diabetes und einer koronaren Herzerkrankung sollten eine Thrombozytenaggregationshemmung, eine ACE-Hemmer-Therapie und eine lipidsenkende Therapie mit Statinen erhalten. Entscheidend für die langfristige Prognose der Herzerkrankungen ist jedoch die frühe Kontrolle der Risikofaktoren für Herzerkrankungen [30].

### Schlaganfall

Der ischämische Schlaganfall gehört ebenfalls zu den vaskulären Folgekomplikationen des Diabetes mellitus, wobei 3 Formen unterschieden werden müssen: Ischämien auf Basis einer zerebralen Mikroangiopathie (z. B. vaskuläre Leukenzephalopathie), arterio-arteriell-embolische Ischämien bei atherosklerotischen Veränderungen der großen und mittelgroßen intra- und extrakraniellen Hirngefäße (einschließlich des Aortenbogens) und kardiale Embolien. Entsprechend den Ergebnissen der INTERSTROKE-Study sind die Faktoren Diabetes, Bluthochdruck, Rauchen, Adipositas, Bewegungsmangel und Vorerkrankungen wie Herzinfarkt oder Vorhofflimmern für 90 % aller Schlaganfälle verantwortlich [31]. Das ausschließlich auf Diabetes zurückführbare Risiko für Schlaganfall beträgt ca. 5–12 %. In der Framingham-Heart-Study ergab sich ein 2,5- bis 3,6-fach erhöhtes Schlaganfallrisiko für Patienten mit Diabetes [5].

## Psychische Folgen des Diabetes

Während die aufgeführten körperlichen Folgekomplikationen als „klassische“ Folgeerkrankungen des Diabetes gelten, werden Depressionen, Angststörungen oder Essstörungen oft als Begleiterkrankungen bezeichnet. Dies ist nur bedingt richtig, da bekannt ist, dass ein nicht geringer Anteil der psychischen Störungen eine Reaktion auf das Leben mit der Erkrankung Diabetes, diabetesbezogenen Stressoren und negativen Folgen des Diabetes (z. B. Akut‑, Folgekomplikationen) darstellt [7, 32]. In einer Vielzahl von Studien konnte gezeigt werden, dass psychische Störungen bei Diabetes gehäuft auftreten und es eine ungünstige Wechselwirkung mit den Anforderungen und der Umsetzung der Diabetestherapie gibt. Zudem gehen psychische Störungen zumeist mit einer schlechteren glykämischen Kontrolle und weiteren Risikofaktoren für Folgekomplikationen sowie einer erhöhten Mortalität einher [7, 8, 33]. Dies betrifft beispielsweise depressive Störungen, Angststörungen, Essstörungen, substanzinduzierte Störungen, somatoforme Störungen, Zwangsstörungen und posttraumatische Belastungsstörungen. Diabetesbezogene Stressoren (Diabetes-related Distress) fördern das Auftreten von psychischen Störungen [34]. Das erhöhte Auftreten von psychischen Erkrankungen und deren negative Auswirkungen auf den weiteren Verlauf des Diabetes legen nahe, psychische Erkrankungen ebenfalls als „Folgeerkrankungen des Diabetes“ zu bezeichnen.

### Depressionen

Menschen mit Diabetes haben im Vergleich zur Allgemeinbevölkerung ein etwa 2‑ bis 3‑fach höheres Risiko, an einer Depression zu erkranken. Daher soll exemplarisch für die verschiedenen psychischen Störungen der Zusammenhang zwischen Diabetes und Depressionen dargestellt werden. In Deutschland gibt es schätzungsweise 800.000–1.100.000 Menschen mit Diabetes und einer komorbiden Depression [35]. Zwischen Diabetes und Depression besteht ein bidirektionaler Zusammenhang: Menschen, die an einer depressiven Symptomatik leiden, weisen eine erhöhte Inzidenz eines Typ-2-Diabetes und Menschen mit Typ-1- und Typ-2-Diabetes ein erhöhtes Risiko für Depressionen auf. Auch bezüglich der Folgeerkrankungen des Diabetes besteht ein bidirektionaler Zusammenhang: Depressionen stellen für Menschen mit Diabetes einen bedeutsamen Risikofaktor für das Auftreten von Folgekomplikationen dar. Andererseits weisen Diabetespatienten mit bestehenden Folgekomplikationen ein erhöhtes Risiko für Depressionen auf [36]. In Metaanalysen ergibt sich ein signifikanter Zusammenhang zwischen Depression und diabetesassoziierten Folgeerkrankungen. Das Depressionsrisiko steigt mit der Anzahl und der Schwere der Folgekomplikationen an, auch akut auftretende Komplikationen gehen mit höheren Depressionsraten einher [7].

Fakt ist, dass das Depressionsrisiko erst nach der Manifestation des Diabetes und dem weiteren Verlauf der Erkrankung ansteigt: Beispielhaft konnten Sun et al. [37] zeigen, dass sowohl subklinische als auch klinische Depressionen bei Menschen mit Prädiabetes und neu diagnostiziertem Diabetes im Vergleich zur Allgemeinbevölkerung nicht erhöht sind, während im weiteren Verlauf der Erkrankung das Risiko für klinische Depressionen um 61 % ansteigt. Das Risiko von subklinischen Depressionen erhöht sich um 11 %. Möglicherweise führt weniger der Status des beeinträchtigten Glukosemetabolismus per se als vielmehr das Wissen um die Diagnose und die Erfahrung von konkreten Belastungen im Zusammenhang mit der Erkrankung zu dem erhöhten Depressionsrisiko. Bei der Entstehung von Depressionen spielen sowohl Stressoren im Zusammenhang mit der Erkrankung, dysfunktionale Bewältigungsfähigkeiten, die das Diabetesselbstmanagement erschweren, aber auch lang andauernder oder sehr intensiver Stress im Leben von Menschen mit Diabetes eine bedeutsame Rolle [38]. Hermanns et al. konnten nachweisen, dass mit einem diabetesspezifischen Interventionsprogramm, welches auf der Basis der kognitiven Verhaltenstherapie auf die Reduktion des diabetesbezogenen Stresses abzielte, sowohl das Auftreten depressiver Symptome als auch einer klinischen Depression reduziert werden konnte [39].

Für die Entstehung von Depressionen spielen bei Diabetes anscheinend die Dysregulation der Hypothalamus-Hypophysen-Nebennierenrinden-Achse und die dadurch bedingten Inflammationsprozesse an den Gefäßen eine zentrale Rolle. Je länger diese homöostatische Dysregulation anhält und je öfter es zu rezidivierenden Verläufen der Depression kommt, desto höher ist im weiteren Verlauf das Risiko von Komplikationen. Dies kann als eine Erklärung für die deutlich erhöhte Mortalität von Menschen mit Diabetes und Depressionen im Vergleich zu Menschen ohne Diabetes dienen [35].

### Kognitive Beeinträchtigungen

Diabetes ist mit einem 1,25- bis 1,91-fach erhöhten Risiko für kognitive Störungen (kognitive Beeinträchtigung und Demenz) verbunden, auch Personen mit Prädiabetes weisen bereits ein höheres Risiko für Demenz auf [40]. Dies ist das Ergebnis der aktuell größten Metaanalyse zu diesem Thema: Mit einem erhöhten Demenzrisiko verbunden sind ein erhöhter Nüchternplasmaglukosespiegel, erhöhte Werte des 2‑Stunden-postprandialen Glukosespiegels, ein erhöhter HbA1c-Wert sowie niedrige und hohe Werte des Nüchternplasmainsulins. Die Wahrscheinlichkeit, dass eine leichte kognitive Einschränkung (LKB oder MCI) in eine Demenz übergeht, ist bei Menschen mit Diabetes im Vergleich zu Personen ohne Diabetes zweifach erhöht [40]. Neben einer genetischen Disposition sind arterielle Hypertonie, chronische Hyperglykämie, komorbide Depressionen, körperliche Inaktivität, Adipositas und Insulinresistenz sowie mikro- und makrovaskuläre Erkrankungen für das erhöhte Demenzrisiko für Menschen mit Diabetes verantwortlich. Hinsichtlich der Entwicklung einer Demenz hat Diabetes im Vergleich zu anderen kardiovaskulären Risikofaktoren einen großen Einfluss – ca. 1 von 10–15 Demenzfällen kann dem Diabetes zugeschrieben werden. Hierbei sind neben Diabetes vor allem vaskuläre Risikofaktoren für die Progression einer vaskulären Demenz verantwortlich [27].

## Lebensqualität

Neben der Vermeidung von Akut- und Folgekomplikationen des Diabetes stellt der Erhalt der Lebensqualität ein wichtiges Ziel der Diabetestherapie dar. Menschen mit Diabetes weisen im Vergleich zur Allgemeinbevölkerung eine deutlich schlechtere Lebensqualität auf [2]. Dies ist auch insofern bedeutsam, als die Befindlichkeit von Menschen mit Diabetes in einem nicht unerheblichen Ausmaß mit der Qualität der Selbstbehandlung und der glykämischen Kontrolle und somit auch mit dem Auftreten von Komplikationen assoziiert ist. Eine geringe Lebensqualität weist auch, unabhängig von somatischen Risikofaktoren, einen prädiktiven Wert für das Auftreten von Folgekomplikationen mit einer erhöhten Mortalität auf. Beispielsweise konnten Khunkaew et al. zeigen, dass ein DFS zu einer deutlich eingeschränkten Lebensqualität führte, was auch mit häufigeren Problemen am Arbeitsplatz, der Mobilität und Selbstversorgung einherging und prognostisch sowohl mit einer höheren Amputationsrate als auch einer erhöhten Mortalität assoziiert ist [41]. Zudem stellt der Umgang mit den Folgeerkrankungen des Diabetes, besonders bei gravierenden Komplikationen, zumeist eine psychische Belastung dar. Häufig treten bei den Patienten auch Schuld- oder Versagensgefühle auf, wenn sie sich der großen Eigenverantwortung bei der Umsetzung der Therapie nicht gewachsen fühlen.

## Prognose des Diabetes

### Mortalität

Anhand der Daten von 65 Mio. bundesdeutschen Versicherten konnte die Exzessmortalität (Übersterblichkeit) bei Diabetes im Jahr 2010 in Deutschland auf 174.627 Menschen abgeschätzt werden. 21 % aller Todesfälle in Deutschland waren auf Diabetes zurückzuführen, 16 % auf Typ-2-Diabetes [42]. Auf der Basis einer Studie mit gesetzlich Krankenversicherten (GKV) in Deutschland kann das altersadjustierte, relative Sterberisiko abgeschätzt werden: Im Vergleich zu Menschen ohne Diabetes ist das Mortalitätsrisiko bei Frauen mit Diabetes um das 1,52-Fache, bei Männern um das 1,56-Fache erhöht. Zwar nimmt das relative Sterberisiko mit zunehmendem Alter ab – allerdings ist es auch im hohen Lebensalter (Alter 80–84 Jahre) bei Frauen noch um das 1,57-Fache und bei Männern um das 1,45-Fache im Vergleich zur Allgemeinbevölkerung erhöht. Bei einem 50-jährigen Mann mit Typ-2-Diabetes sinkt die Lebenserwartung um ca. 6 Jahre, bei einer 50-jährigen Frau um ca. 6,5 Jahre [43]. Die Mortalität ist sowohl für makro- als auch mikrovaskuläre Erkrankungen im Alter reduziert: Jedes um ein Jahr höhere Alter bei der Diabetesdiagnose war – jeweils bereinigt um das aktuelle Alter – mit einem um 4 %, 3 % bzw. 5 % geringeren Risiko für die Gesamtmortalität, makrovaskuläre und mikrovaskuläre Erkrankungen verbunden [44]. Die schlechteste Prognose haben offensichtlich Kinder, Jugendliche und junge Erwachsene, die an Typ-2-Diabetes erkranken, obwohl hier noch keine Mortalitätsdaten vorliegen. Die Ergebnisse der TODAY-Studie, die 500 Teilnehmer mit einer mittleren Zeit seit der Diabetesdiagnose von 13,3 ± 1,8 Jahren (Alter: 26,4 ± 2,8 Jahre) beobachtete, zeigen, dass bereits nach dieser vergleichsweise kurzen Zeitspanne 67,5 % der Teilnehmer Bluthochdruck, 51,6 % Dyslipidämie, 32,4 % diabetische Nierenerkrankung/Nervenerkrankungen und 51,0 % Netzhauterkrankungen, einschließlich fortgeschrittener Stadien aufweisen. Mindestens eine Komplikation trat bei 60,1 % der Teilnehmer auf, bei 28,4 % traten mindestens 2 Komplikationen auf. Hauptrisikofaktoren für die Entwicklung von Komplikationen waren vor allem die Zugehörigkeit zu einer Minderheit oder ethnischen Gruppe, Hyperglykämie, Bluthochdruck und Dyslipidämie [45].

### Steigende Lebenserwartung und Lebensjahre mit gesundheitlichen Einschränkungen

Elliott Joslin, der Nestor der Diabetologie, schrieb in einem Mission-Statement im Jahr 1923, nachdem Insulin zunehmend weltweit verfügbar war, dass Kinder mit Typ-1-Diabetes zukünftig ein gesundes und langes Leben wie Menschen ohne Diabetes führen können. Allerdings schätzte er bereits 1940 für ein 10-jähriges Kind mit neu diagnostiziertem Diabetes eine Anzahl von 40 weiteren Lebensjahren im Vergleich zu 57 Jahren für ein Kind ohne Diabetes desselben Alters. Eine retrospektive Analyse von Patientendaten der Joslin-Klinik aus den Jahren 1923 bis 1945 zeigte später, dass Patienten mit einer Diagnose vor dem Alter von 30 Jahren im mittleren Alter von 44 Jahren starben, was einem Verlust von 25 Lebensjahren im Vergleich zur Normalbevölkerung entsprach. Eine ähnliche Analyse von Patientendaten aus dem Jahre 1959 erbrachte einen Verlust von 20 Lebensjahren [46].

Mittlerweile zeigt eine Reihe von Studien, dass sich die Lebenserwartung von Menschen mit Typ-1- und Typ-2-Diabetes in den letzten Jahrzehnten bedeutend erhöht hat. Laut den Ergebnissen einer Metaanalyse konnte im Vergleich der Zeiträume 1990–1999 und 2000–2016 in 75 % aller europäischen Länder und in 57 % der nichteuropäischen Länder ein Rückgang der Sterblichkeitsrate beobachtet werden. Dieser Rückgang war zumeist stärker ausgeprägt als bei der Allgemeinbevölkerung, was als ein Hinweis auf verbesserte Versorgungsbedingungen für Menschen mit Diabetes gewertet werden kann, obgleich das Mortalitätsrisiko noch immer erhöht ist [9].

Die höhere Lebenserwartung führt allerdings auf der anderen Seite zu einem Anstieg der Lebensjahre mit gesundheitlichen Einschränkungen, also einer Verringerung der Healthy Life Years (HLY). Die Auswertung des nationalen GEDA-Surveys (2009–2012) ergab bei Personen mit Diabetes im Vergleich zu Personen ohne Diabetes für beide Geschlechter eine deutlich geringere Anzahl gesunder Lebensjahre. Dies gilt besonders für Menschen im jüngeren und mittleren Lebensalter: Bei 30- bis 34-Jährigen mit Diabetes ist die erwartete gesunde Lebenszeit für Frauen 11 Jahre und für Männer 12 Jahre geringer als in der Allgemeinbevölkerung. Diese Differenz verändert sich mit zunehmendem Alter. So betrug der Unterschied in der Altersgruppe 70 bis 74 Jahre bei Frauen 4,3 Jahre und bei Männern 3,4 Jahre, was aber nach wie vor einen beträchtlichen Unterschied darstellt [47].

### Langzeitprognose: Ergebnisse großer Interventionsstudien

Zwei große Landmark-Diabetesstudien lieferten wichtige Hinweise, welche Faktoren für das Langzeitüberleben verantwortlich sind: die amerikanische DCCT (Diabetes Control and Complications Trial) von 1983–1993 zum Vergleich einer konventionellen und intensivierten Insulintherapie bei Diabetes-Typ‑1 mit 1441 Teilnehmern [48] und die englische UKPDS (UK Prospective Diabetes Study) von 1977–1997 zum Vergleich der Therapie mit Sulfonylharnstoffen, Metformin und Insulin bei Typ-2-Diabetes mit 5102 Teilnehmern [49].

Die Gesamtmortalitätsrate in der DCCT im 27-Jahre-Follow-up zwischen den beiden Behandlungsgruppen unterschied sich erst nach 15 Jahren der Nachbeobachtung mit einem Vorteil für die intensivierte Insulintherapie (HR = 0,67 [95 % KI, 0,46–0,99]; *p* = 0,045). Insgesamt waren Teilnehmer der DCCT, die zum Follow-up verstarben, älter, wiesen ein höheres Alter bei Diabetesbeginn auf, waren eher Raucher und hatten höhere systolische Blutdruck‑, Cholesterin- und HbA1c-Werte, Albuminurie und Nierenerkrankung im Endstadium. Die häufigsten Todesursachen waren kardiovaskuläre Ereignisse (22,4 %), Krebs (19,6 %), Akutkomplikationen des Diabetes (17,8 %) und Unfälle oder Suizid (16,8 %). Im Studienarm mit der intensivierten Insulintherapie gab es gegenüber der konventionell behandelten Gruppe signifikant weniger Todesfälle durch Nephropathie, kardiovaskuläre Erkrankungen und Krebs [50].

In der UKPDS hatte eine strikte Glukosekontrolle zu Beginn der Therapie des Typ-2-Diabetes ab dem Zeitraum von 10 Jahren Nachbeobachtung unabhängig von den Treatmentbedingungen einen positiven Effekt hinsichtlich der Gesamtmortalität – dies wird als „Legacy-Effekt“ bezeichnet. In einer aktuellen Publikation konnten Lind et al. zeigen, dass ein um 1 % höherer HbA1c-Wert in den ersten Jahren der Behandlung mit einem zunehmend steigenden Sterberisiko einhergeht: 8 % (5 Jahre), 18 % (10 Jahre), 28 % (15 Jahre), 36 % (20 Jahre). Bei einem um 2 % erhöhten HbA1c-Wert betrug die Erhöhung des Mortalitätsrisikos 16 % (5 Jahre), 40 % (10 Jahre), 64 % (15 Jahre), 86 % (20 Jahre). Die Autoren schlussfolgern, dass historische Glukosewerte, vor allem in der Phase der ersten Jahre der Behandlung, die Prognose des Typ-2-Diabetes deutlich stärker beeinflussen als die nachfolgenden bzw. aktuellen Glukosewerte, und fordern eine frühe Diabetesdiagnose und eine möglichst normnahe Glukosekontrolle vor allem zu Beginn der Diabetestherapie [51].

## Fazit und Ausblick

Nach wie vor besteht bei Diabetes ein erhöhtes Risiko, an physischen wie psychischen Folgen des Diabetes zu erkranken. Auch sind die Lebensqualität und -erwartung geringer als bei der nicht diabetischen Vergleichsgruppe. Allerdings gibt es Hinweise, dass durch bessere Versorgungsstrukturen und neue Technologien, digitale Anwendungen in der Forschung und Therapie dieser Unterschied zukünftig geringer wird. Es bleibt zu hoffen, dass wir in Zukunft mit Ansätzen der Präzisionsmedizin [52] noch besser die individuellen Risiko- wie auch protektiven Faktoren für die Entstehung von Folgekomplikationen des Diabetes kennen, um diese besser vermeiden zu können.
